# Vanillin as an Antifouling and Hydrophilicity Promoter Agent in Surface Modification of Polyethersulfone Membrane

**DOI:** 10.3390/membranes9040056

**Published:** 2019-04-24

**Authors:** Mohammadamin Esmaeili, Tiina Virtanen, Jussi Lahti, Mika Mänttäri, Mari Kallioinen

**Affiliations:** 1 LUT School of Engineering Science, Department of Separation and Purification Technology, LUT University, 53850 Lappeenranta, Finland; tiina.virtanen@lut.fi (T.V.); jussi.lahti@lut.fi (J.L.); mika.manttari@lut.fi (M.M.); mari.kallioinen@lut.fi (M.K.); 2 LUT Re-Source Platform, LUT University, P.O. Box 20, 53851 Lappeenranta, Finland

**Keywords:** polyethersulfone, vanillin, hydrophilicity promoter, antifouling

## Abstract

Fouling as an intricate process is considered as the main obstacle in membrane technologies, and its control is one of the main areas of attention in membrane processes. In this study, a commercial polyethersulfone ultrafiltration membrane (MWCO: 4000 g/mol) was surface modified with different concentrations of vanillin as an antifouling and hydrophilicity promoter to improve its performance. The presence of vanillin and its increasing adsorption potential trends in higher vanillin concentrations were clearly confirmed by observable changes in FTIR (Fourier transform infrared) spectra after modification. Membranes with better hydrophilicity (almost 30% lower contact angle in the best case) and higher polyethylene glycol solution (PEG) permeability were achieved after modification, where a 35–38% increase in permeability of aqueous solution of PEG was perceived when the membrane was modified at the highest exposure concentration of vanillin (2.8 g/L). After filtration of wood extract, surface modified membrane (2.8 g/L vanillin) showed better antifouling characteristics compared to unmodified membrane, as indicated by approximately 22% lower pure water flux reduction, which in turn improved the separation of lignin from the other organic compounds present in wood extract.

## 1. Introduction

One of the most substantial polymeric materials which is extensively exploited in the field of membrane separation technology is polyethersulfone (PES). A considerable attention has been placed on PES–based membranes due to their appropriate mechanical properties, superior thermal strength and excellent oxidative and hydrolytic stability [1]. Furthermore, PES is applied as the main base material for membranes in several membrane separation processes like haemodialysis, purification, extraction and concentration [2]. Despite its many attractive properties, one of the key problems to the widespread use of PES membranes is the proneness to fouling because of its low hydrophilicity [3]. For example, several studies have reported severe fouling tendency of PES-based membrane in the treatment of process waters from the pulp and paper industry, and the biorefinery streams [4,5]. Consequently, the development of structures and advanced materials in order to decrease the impact of fouling and biocompatibility have been in focus in recent research in the field of membrane technology. State-of-the-art membrane materials are relatively high-priced; thus, the modification of commercial membranes could be an appropriate alternate method in order to develop membranes with improved biocompatibility and better antifouling characteristic and functionality [6,7].

Surface modification via physical adsorption is a simple, cheap and flexible tool for modifying and structuring polymer surfaces, and it has been widely adopted to tailor the surface properties of different kind of polymeric membranes [8]. This technique is generally used for three main reasons: (1) need to increase hydrophilicity, (2) need to reduce surface roughness, and (3) need to optimize the surface charge of the membrane. For example, Reddy et al. [9] used poly(sodium 4-styrenesulfonate) (PSS) as a negatively charged polymer for surface modification of commercial PES ultrafiltration membranes with different cut-off values. They demonstrated that membranes with better antifouling characteristics and better rejection of salts and polyethylene glycol (PEG), can be obtained by permeation of aqueous solution of the PSS through the membrane [9]. Zhang et al. studied the surface modification of commercial Reverse Osmosis (RO) membrane, and suggest that commercial alkaline lignin deposited on the surface of the RO membrane improved the salt rejection, the fouling resistance and the water permeation of the membrane [10].

Surface modification of membranes with natural components possessing quorum sensing inhibitory, and hydrophilicity promoting characteristics can be considered as a novel physical modification paradigm, which can improve the membrane performance and also inhibit the biofilm formation and bacterial growth, which in turn decreases membrane biofouling. Vanillin, a potential hydrophilic additive, has both polar and non-polar structural characteristics and has been widely used as a flavoring substance in food. Due to its antioxidant and antimicrobial features, it has potent preservative characteristics similar to most phenolic compounds and thus can be used in the food industry [11]. The quorum sensing inhibition (QSI) behavior of vanillin has been demonstrated in several studies in membrane-based applications [12,13,14,15,16]. In a study by Katebian et al. [16], the inhibitory characteristics of vanillin as QS agent against biofilm formation of a mixture of marine bacteria was evaluated by physical adsorption of vanillin on the surface of polyamide thin-film RO membranes. It was demonstrated that the disruption of QS pathways reduced the biomass production of polysaccharide (∼12%), live bacteria (∼58%) and dead bacteria (∼16%) on the surface of the membrane (SWC5, Oceanside, CA), and as a result it reduced the biofilm formation. However, no significant improvement in membrane performance, i.e., salt rejection and pure water permeability, was observed [16].

In this study, the surface modification of commercial polyethersulfone ultrafiltration (UF) membrane (UH004 P, Microdyn-Nadir GmbH, Wiesbaden, Germany) with controlled adsorption of different concentrations of vanillin as an antifouling and hydrophilic promoter agent was explored in order to improve the performance of the membrane. The performance of unmodified membrane and vanillin modified membranes were evaluated based on the permeability and rejection of aqueous solution of polyethylene glycol. Herein, we also characterized the characteristics of membranes via Fourier transform infrared spectroscopy (FTIR), contact angle measurements before and after precleaning with Ultrasil 110 (chemical-cleaning agent, Ecolab, St. Paul, MN, USA) and water, and after surface modification. In addition, the quantity of vanillin, which remained in the membrane structure after experiment, was measured by extraction of vanillin from membrane samples and subsequent UV analysis. The antifouling characteristics of the best modified membrane in terms of membrane performance were also appraised, by measuring the pure water flux before and after the filtration of pressurized hot water extract (PHWE) and comparing to virgin membrane.

## 2. Materials and Methods

### 2.1. Chemicals

Commercial PES UF membrane UH004 P with nominal molecular weight cut-off (NMWCO) of 4 kDa obtained from Microdyn-Nadir GmbH, Wiesbaden, Germany, was utilized as a base membrane for both membrane surface modification and fouling experiment. The model compound utilized in rejection study was Polyethylene glycol (PEG, CAS: 25322-68-3) with molecular weight of 4 kD purchased from Fluka AG (Buchs, Switzerland). Vanillin with a purity of 99% (Mw. 152.15 g/mol, CAS: 121-33-5) and hydrochloric acid with European Pharmacopoeia quality standard (HCl, 37.0%, CAS: 7647-01-0), were provided by Acros Organics (Geel, Belgium) and VWR chemicals, respectively. Methanol (J. T. Baker, the Netherlands, CAS: 67-56-1) was used as the solvent for extraction of vanillin from the surface of the membrane. Ultra-pure deionized water (DI, 0.5–1 μS/cm at 25 ${}^{\circ }$C) was obtained from CENTRA-R 60/120 system (Elga purification system, Veolia Water, Bucks, UK) and was used for all experiments and preparation of solutions.

### 2.2. Membrane Characterization

#### 2.2.1. Fourier Transform Infrared Spectroscopy

The PerkinElmer Frontier spectrometer (Perkin-Elmer Inc., Waltham, MA, USA) with universal ATR (attenuated total reflection) module (diamond crystal) was used to analyse the FTIR spectra of virgin and modified PES membranes in order to illustrate the presence of vanillin in modified PES membranes. A spectral resolution of 4 cm${}^{-1}$ and wavenumber range of 4000–400 cm${}^{-1}$ were used in measuring the FTIR spectra of each membranes from six random spots. At the absorbance mode, 20 scans of 1 cm${}^{-1}$ data interval were acquired for all the spectra. Finally, ATR correction, baseline correction and normalization were utilized in processing the co-added spectra.

#### 2.2.2. Contact Angle Measurement

Static contact angle (CA) of membranes before and after precleaning with the Ultrasil 110 cleaning agent and also after modification with different concentrations of vanillin solution were measured based on captive bubble method. Using a U-shaped needle at room temperature, an air bubble (roughly a 3–4 μL) was placed on the surface of each membrane sample. For each sample, the contact angle was measured at six independent points and the average taken as the actual CA for that sample. This was done to increase measurement reliability. The KSV CAM 101 instrument (KSV Instruments Ltd., Helsinki, Finland) coupled with a DMK 21F04 CCD camera (The Imaging Source Europe GmbH, Bremen, Germany) was used to measure the CA of each sample. To determine the contact angle, curve fitting analysis of the captured images was processed using the CAM 2008 software (KSV Instruments Ltd., Helsinki, Finland).

#### 2.2.3. Surface Charge Measurements

One of the most important factors in aqueous membrane filtration is the surface charge which is also associated to the zeta potential. An electro-kinetic analyzer (SurPASS, Anton Paar, Graz, Austria) using 1 mM KCl solution as the background electrolyte and, having an adjustable gap cell, was used to measure the zeta potential of the membrane samples. Before the measurements, pieces of the membrane were immersed in deionized (DI) water and stored in a refrigerator of about 4 ${}^{\circ }$C. Dilute KOH solution was first used to increase the solution pH to 8, then, 0.05 M HCl solution was used during the analysis to decrease the pH of the solution to 3. Lastly, according to the classic Helmholtz–Smoluchowski equation [17] presented in Equation (1), the streaming current measurement was applied in calculating the zeta potential (ζ (V)).

(1) $$\zeta =\frac{E_{S}}{\Delta P}\frac{\eta \kappa }{\varepsilon_{r}\varepsilon_{0}},$$

where *E_s_* is the tangential streaming potential (V), Δ*P* the pressure gradient (Pa), κ the conductance of the electrolyte solution (S·m${}^{-1}$), η liquid viscosity (kg·m${}^{-1}$·s${}^{-1}$), ε${}_{0}$ vacuum permittivity (F·m${}^{-1}$), and ε${}_{r}$ is the relative dielectric constant (dimensionless).

#### 2.2.4. Extraction of Vanillin from Membrane and UV Analysis

UV spectrophotometer (Jasco V-670 spectrophotometer, Tokyo, Japan) was utilized to measure the concentration of lignin in feed, permeate, and concentrate by UV absorption at the maximum wavelength of 280 nm, and also to determine the amount of adsorbed vanillin on the surface of commercial membranes after physical modification tests. The amount of residual vanillin on the surface and structure of the modified membranes after tests was determined according to the extraction procedure that was presented in detail by Virtanen et al. [18]. Due to the possible presence of vanillin in the permeate of PEG rejection experiments, six different concentrations of vanillin were prepared at pH 5.6 (the pH of permeates in PEG experiments) and at 25 ${}^{\circ }$C in order to determine the molar adsorption coefficient (ε) of vanillin in water (Figure S3, Supplementary Material). This coefficient was used in correction of PEG rejection calculations, which might be affected by the presence of vanillin in permeate.

### 2.3. Experimental Design and Procedure

#### 2.3.1. Preparation of Pressurized Hot Water Extract

The autohydrolysis process was performed in a batch extractor set up. The pine wood saw dust with particle size distribution below 2.8 mm was used to prepare wood extract. A 5.6:1 weight ratio of water to wood was applied into 15 L stainless steel reactor, and then heated to 160 ${}^{\circ }$C and processed for 90 min at pressure of 5–6 bar. The extract was cooled down to 25 ${}^{\circ }$C followed by 30 min centrifugation at 3000 rpm (Sorvall RC-28S centrifuge, RCF = 920G GSA fixed angle rotor, Du Pont, Wilmington, DE, USA). The supernatant was meticulously separated from insoluble sediment for fouling studies. The pH and conductivity of the PHWE was 3.8 and approximately 600 μS/cm at 25 ${}^{\circ }$C.

#### 2.3.2. Surface Modification of PES Membrane with Vanillin

**Figure 1:**
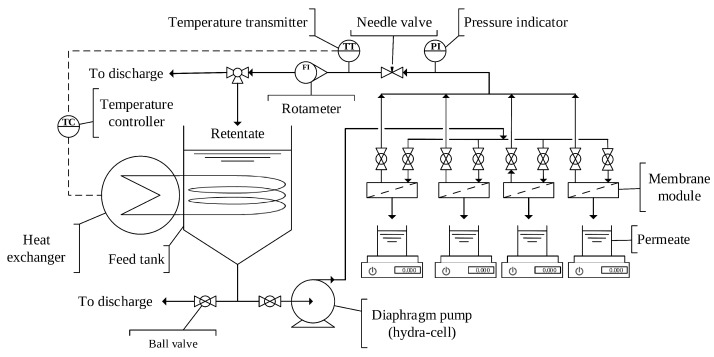
A schematic diagram of cross-flow laboratory-scale filtration system.

Commercial polyethersulfone membrane was utilized in order to study adsorptive surface modification of membrane, rejection of PEG and fouling caused by wood extract. All the membranes were initially precleaned with Ultrasil 110 cleaning agent (0.2%, pH 13.6) for 30 min prior to use, in order to eliminate both the preservative agent and also any impurities existing on the surface of the membrane. Following this treatment, the membrane coupons were soaked in DI water and stored in a refrigerator overnight at 4 ${}^{\circ }$C for completing membrane wetting. The rejection study of PEG was conducted by using a four- parallel rectangular cross-flow flat-sheet modules which were made of stainless steel (AISI 316). The schematic configuration of the filtration system unit combined with heat exchanger is depicted in Figure 1. The dimensions used to construct the channel height, length, and width of each module were 0.1 cm, 10.0 cm, and 4.0 cm, respectively. The effective surface area of the membrane was 40 cm${}^{2}$. In every experiment, the temperature was monitored using an inline thermometer probe and it was adjusted at 25 ± 1 ${}^{\circ }$C by using a heat exchanger (Lauda Proline RP 855 thermostat, Lauda-Königshofen, Germany).

To begin the rejection studies, in all experiments, the membrane coupons were mounted in the module and the filtration system was flushed with DI water, without applying pressure until the conductivity of the DI water was less than 3 μS/cm. Afterwards, the pressure was increased to 3 bar by using a needle valve and both membrane and the system were flushed at 25 ${}^{\circ }$C with the same criteria as in previous step. During the flushing process, the cross-flow velocity was measured by rotameter and adjusted to 0.65–0.7 m/s for each membrane cell, using pump (Hydra-cell model M-03, Wanner Engineering, Inc., Minneapolis, MN, USA) equipped with Vacon frequency converter, which was used to control the flow with the pump. Once the membranes and system were appropriately washed, the membranes were pressurized at 5 bar for 45 min to minimize the effect of compaction phenomenon and also to remove the possible leftover preservative agent. Thereafter, the pressure was reduced to 3 bar and membrane flux was stabilized for 15 min before measuring the PEG rejection and permeability. Then, the flux of 1 g/L PEG solution for each membrane was measured under 3 bar with the interval of 2 min until the relative steady flux was obtained (three successively recorded values gave the same result with the standard deviation of 0.1). Once the steady flux was achieved, the permeate of each membrane as well as the feed solution was collected to calculate the apparent PEG rejection using Equation (2):

(2) $$R\left(\%\right)=\left(1-\frac{C_{p}}{C_{f}}\right)\times 100,$$

where *R*, *C*${}_{p}$ and *C*${}_{f}$ are PEG rejection, the concentration of PEG in downstream (permeate) and concentration of PEG in upstream (feed), respectively.

The total organic carbon (TOC) was considered as a detection index to measure the concentration of PEG. For this purpose, TOC analyzer (Shimadzu TOC-L series, Kyoto, Japan) equipped with a non-dispersive infrared (NDIR) detector was utilized. Prior to commencing the surface modification of membrane with vanillin, both system and membrane were flushed with the aforementioned procedure. The surface modification of membrane was accomplished with six vanillin concentrations at pH 3.8: 0.3 g/L, 0.8 g/L, 1.3 g/L, 1.8 g/L, 2.3 g/L, and 2.8 g/L (pH was adjusted using 0.1 M hydrochloric acid solution). DI water at pH 3.8 was considered as a reference to evaluate the effect of pH on the permeability of PEG.

Vanillin solutions at pH 3.8 were recirculated over the surface of the membrane without applying pressure for 80 min using peristaltic pump (Masterflex^®^ L/S^®^ Model: 7519-05 Cartridge Pump heads, Cole-Parmer International, Vernon Hills, IL, USA) at 25 ${}^{\circ }$C. The peristaltic pump was equipped with four identical cartridges and it was programmed to supply 300 ± 10 g/min at 25 ${}^{\circ }$C to each membrane module through separated flow lines from each of reservoirs.

The membranes were subsequently flushed with DI water to remove any unattached vanillin from the surface of membranes. Then, after 15 min stabilization at 3 bar, the flux of PEG solution with the same above-mentioned procedure was measured for all membranes. Finally, the membranes and filtration system were flushed with DI water, and the membrane coupons were taken out from the modules and dried at room temperature for 24 h for further UV and IR analysis.

To assess whether and how vanillin can effect on the hydrophilicity of the membrane and also to evaluate the amount of adsorbed vanillin on the surface of the membrane, another test set was also designed. In this experiment set, after flushing the system and membrane with the same aforementioned criteria and conditions, the membrane coupons were pressurized at 5 bar and at 25 ${}^{\circ }$C for 45 min and then with the same procedure vanillin solutions were adsorbed on the surface of the membrane. Finally, modified membrane coupons were directly taken out from the modules and dried overnight (24 h) at room temperature for further FTIR and contact angle analyses.

#### 2.3.3. Fouling Study of the Virgin and Modified Membranes with Wood Extract

**Figure 2:**
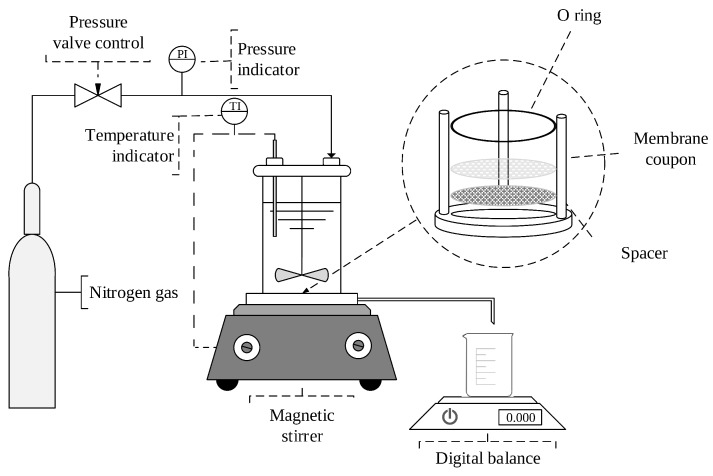
Schematic configuration of the dead-end filtration system.

The fouling behavior and permeance of wood extract for both virgin and modified membranes was investigated in a batch mode with a dead-end filtration system using Amicon solvent-resistance stirred cell (Millipore, Merck KGaA, Darmstadt, Germany, Cat No.: XFUF07611, diameter of mixer: 60 mm). The schematic diagram of dead-end filtration unit is presented in Figure 2.

Commercial membranes with a diameter of 76 mm (active diameter of 70 mm) were initially prepared and then precleaned using the same procedure as explained in Section 2.3.2. As a preliminary step, the compaction of membranes at 5 bar for 20 min and subsequent 15 min stabilization at 3 bar with 300 mL DI water was conducted. Then, pure water flux at 3 bar and 25 ${}^{\circ }$C was measured before static adsorption of vanillin on the surface of the membrane. Afterwards, 300 mL of vanillin solution with concentration of 2.8 g/L was added into the Amicon cell, and the outer surface of the membrane was exposed for 80 min without any applied pressure at a stirring rate of 500 rpm. Thereafter, 300 g centrifuged wood extract was filtered for 90 min and the filtrate weight was recorded every one minute. At the end of the filtration test, the final weight of permeate and concentrate were recorded in order to evaluate the mass balance. Then, the membrane coupon was taken out and the surface of the membrane was rinsed six times by dipping it into the DI water. Finally, the pure water flux was measured with the same conditions which was performed before static adsorption step. The pure water flux measurement proceeded until three sequentially recorded weights remained constant. In the case of virgin membrane, the same procedure was implemented except for exposure of membrane surface with vanillin. The lignin and TOC rejections were calculated using Equation (3):

(3) $$R\left(\%\right)=\left(1-\frac{2\times c_{p}}{c_{f}+c_{r}}\right)\times 100,$$

where *C*${}_{p}$, *C*${}_{f}$ and *C*${}_{r}$ are the concentration of lignin or TOC in permeate, initial feed (i.e., *t* = 0) and retentate (at the end of filtration process), respectively. The concentration of lignin was calculated from Beer–Lambert law by using the absorption coefficient (i.e., absorptivity) of pine lignin at 280 nm, *ε* = 25 L·g${}^{-1}$·cm${}^{-1}$ [19,20]. Membrane fouling was evaluated based on pure water flux reduction (PWF${}_{R}$) using Equation (4):

(4) $${\mathrm{PWF}}_{R}\left(\%\right)=\left(\frac{{\mathrm{PWF}}_{b}-{\mathrm{PWF}}_{a}}{{\mathrm{PWF}}_{b}}\right)\times 100,$$

where PWF${}_{b}$ (kg/m${}^{2}$h) and PWF${}_{a}$ (kg/m${}^{2}$h) are pure water mass fluxes before and after the filtration of PHWE.

## 3. Results and Discussion

### 3.1. Characterization of Membranes by the Means of FTIR Spectroscopy

#### 3.1.1. Spectral Analysis of Virgin Membranes

**Figure 3:**
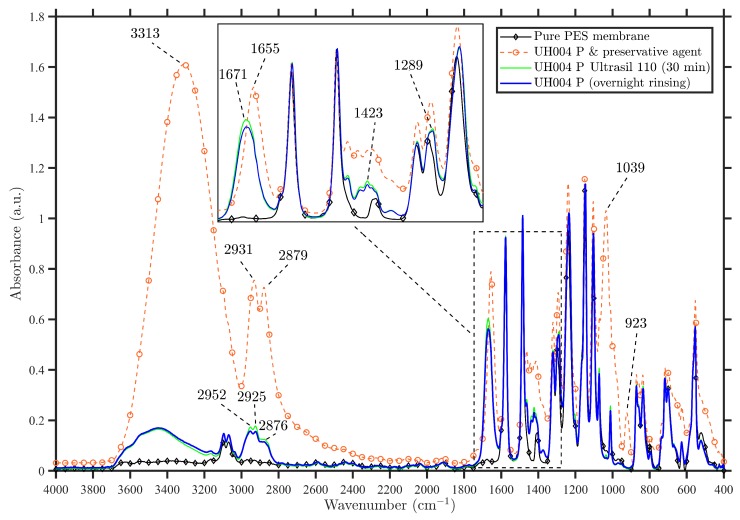
The effect of overnight rinsing with deionized (DI) water and 30 min precleaning with Ultrasil 110 cleaning agent on the ATR-FTIR spectra of the UH004 P membrane in the region of 4000–400 cm${}^{-1}$.

The ATR-FTIR spectra of the unmodified UH004 P membrane over the wavenumber range from 4000 to 400 cm${}^{-1}$, before any precleaning steps, after overnight rinsing with DI water, and after 30 min precleaning with Ultrasil 110 cleaning agent, are presented in Figure 3. According to the acquired spectra presented in Figure 3, the presence of preservative agent on the surface of UH004 P membrane before any precleaning can be clearly observed, due to a very strong absorption band at 3313 cm${}^{-1}$, and three other bands at 1655, 1039, 923 cm${}^{-1}$. Accordingly, these peaks relate to the presence of a preservative agent [21,22], which is consistent with the FTIR spectrum of glycerol obtained in earlier studies [23,24,25]. Hence, glycerol is the preservative agent in the unmodified UH004 P membrane.

The main functional groups of glycerol are presented in Table 1, which disappeared completely after precleaning of membrane samples with DI water (overnight rinsing), and also after precleaning with the Ultrasil 110 cleaning agent. As can be seen from Figure 3, the same results with no perceptible difference are obtained after overnight rinsing with DI water and after 30 min precleaning with the Ultrasil 110 cleaning agent. The reason for this similarity can be attributed to the hydrogen bonding of three hydroxyl groups of glycerol with water resulting in higher solubility of this compound in water.

A comparison of the UH004 P membrane spectra after precleaning with the spectrum of pure PES membrane (without any additives) reveals some unique peaks demonstrating the fact that the commercial membrane has been modified with additive(s). To the authors’ knowledge, UH004 P membrane might be modified with polyvinyl pyrrolidone (PVP). Comparing the additional peaks which are highlighted as PVP bands in Table 2 reinforces this hypothesis.

Two signals at 1486 and 1578 cm${}^{-1}$ are identical peaks for PES membranes [21,27]. Considering the fact that there is always a little amount of inevitable trapped water inside the porous membranes, fabricated via phase inversion techniques, the presence of two peaks at 3553 and 3636 cm${}^{-1}$ can be attributed to O–H stretching vibration of water molecules, which is in line with the earlier studies [21].

#### 3.1.2. Spectral Analysis of the Commercial Membrane Modified by Vanillin

**Figure 4:**
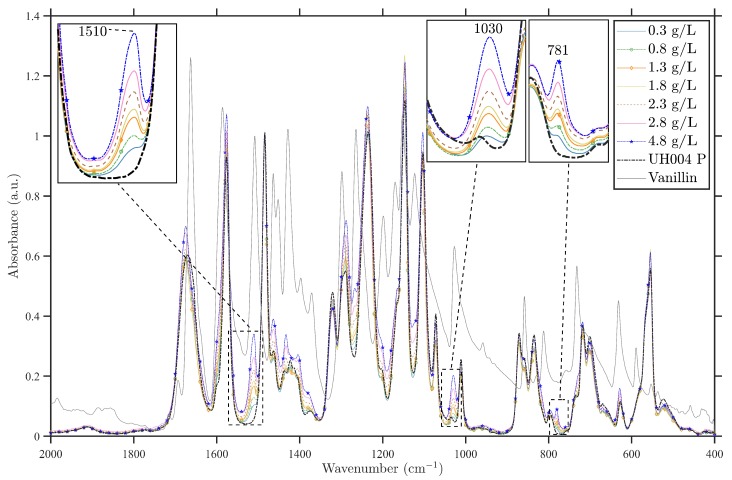
FTIR spectra of virgin and modified UH004 P membranes for various concentrations in the region of 2000–400 cm${}^{-1}$. The peaks corresponding to the presence of vanillin can be distinguished at 1510, 1030 and 781 cm${}^{-1}$.

The results obtained from the FTIR analysis of the virgin UH004 P and the modified ones at different vanillin concentrations are presented in Figure 4, over the range from 2000 to 400 cm${}^{-1}$. From the spectrum shown in Figure 4, it can be seen that most of the vanillin peaks overlap with the peaks of the UH004 P membrane due to similarities in their functional groups. Closer inspection of the figure shows a clear trend of increasing intensity of vanillin peaks as a function of vanillin concentration at 1510, 1267, 1030 and 781 cm${}^{-1}$. It is worth emphasizing that the intensity of mentioned peaks increases as the concentration of adsorbed vanillin rises, which is consistent with findings obtained by Virtanen et al. [18]. In addition, it can be noticed that the intensity of some UH004 P membrane peaks rise, as the concentration of adsorbed vanillin increases (e.g., peaks at 1577, 1464, 1288 and 1267 cm${}^{-1}$).

The unique bands for vanillin at 781, 1030, 1267, 1509 cm${}^{-1}$ are associated with aromatic native structure of vanillin, in–plane C–H deformation, OCH${}_{3}$ group, and the coupled skeletal vibrations of aromatic ring of vanillin, respectively [30]. The area of CH–aromatic stretching vibration (3100–3000 cm${}^{-1}$) and aliphatic stretching vibration (2922–2875 cm${}^{-1}$) becomes also more intense as the vanillin concentration increases.

### 3.2. The Effect of Modification on the Membrane Performance

The permeabilities of PEG solution as a function of time, before and after the physical adsorption of vanillin on the surface of the UH004 P membrane, are plotted in Figure S1 (Supplementary Material) and Figure S2 (Supplementary Material). Since the heterogeneity of commercial membranes is not always the same, especially in experiments with a small membrane area, the average increase of permeabilities are presented in Table 3. It is apparent that the permeability increases as the vanillin concentration in the solution rises from 0 to 1.3 g/L. There were no significant changes observed after 1.3 g/L vanillin concentration, which can be explained based on the hydrophilicity of membrane evaluated with respect to contact angle (Table 4). However, a 35–38% increase in permeability was perceived at the highest exposure concentration (2.8 g/L).

### 3.3. The Effect of Modification on the Contact Angle of the Membrane

The contact angles of the precleaned membrane with Ultrasil 110 cleaning agent, and modified membranes with various concentrations of vanillin were measured based on captive bubble method and are presented in Table 4. The results, as shown in Table 4, indicate a positive correlation between successive increase in concentration of vanillin (until 1.8 g/L) and hydrophilicity of the UH004 P membrane. Data from this table can be compared with the data in Table 3, which shows the improvement of permeability as vanillin concentration was increased from 0 to 1.3 g/L. The contact angles remained approximately constant as vanillin concentration was raised from 1.3 to 2.3 g/L, which is in accordance with improvement (%) of PEG solution permeabilities in this range. In addition, it can be noticed that the contact angle of the UH004 P membrane in the presence of glycerol is lower than the contact angle of the precleaned one, due to the presence of three hydroxyl groups in glycerol, which makes the surface more hydrophilic.

From Table 4, it can also be seen that the exposure of the membrane with vanillin concentration above 2.3 g/L results in the increase of the contact angle. This rise might be attributed to the surface roughness. A recent study by Lin et al. [31] shows that the UH004 P membrane has a smooth surface with root-mean-squared roughness (R${}_{rms}$) of 2.8 nm. Therefore, it seems that, at vanillin concentration higher than 2.3 g/L, the surface roughness amplifies the hydrophobicity, which can lead to increase of apparent contact angle [32]. The air traps between the rugosities of solid surface and the liquid droplet, result in larger contact angle for rough surfaces compared to smooth surfaces [33].

**Figure 5:**
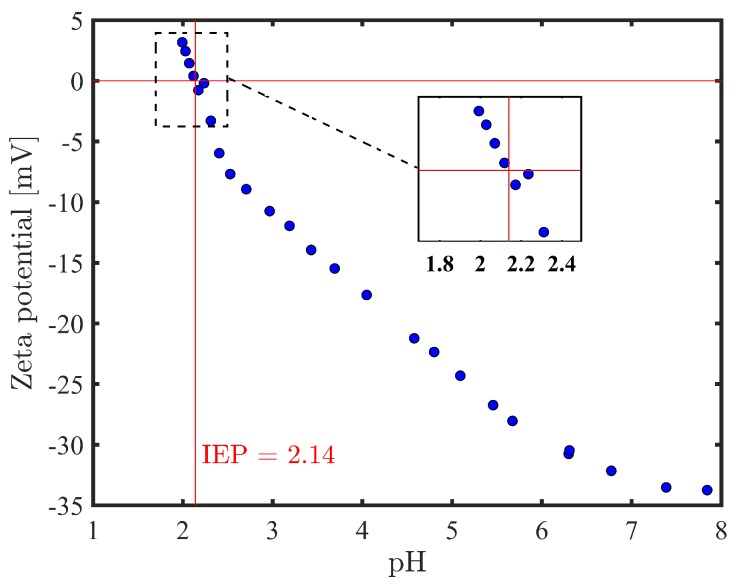
Zeta potential versus pH for UH004 P membrane without preservative agent.

Lin et al. demonstrated that the UH004 P membrane possesses negatively charged surface over the wide pH range (2–10) which increases with successive rise of pH [31]. The zeta potential versus pH of the UH004 P membrane without preservative agent was measured with a SurPASS electrokinetic analyzer (Anton Paar GmbH, Graz, Austria). As can be seen from Figure 5, the overall observations are consistent with data obtained by Lin et al. [31] while the isoelectric point (IEP) was reached at approximately pH 2.14. As can be seen from the Table 3, the permeability of PEG solution for unmodified membrane (virgin, no vanillin) decreases when the UH004 P membrane is treated with water at pH 3.8, and this might be as a result of surface charge reduction of the UH004 P membrane. Moreover, ethylene units in PEG molecules prefer gauche conformation due to hydrophobic interactions [34]. Partially negatively charged ether groups of PEG interact with water molecules and are thus exposed and interact with the surface of the membrane. Reduction in surface charge of the membrane amplifies the flux reduction of PEG solution, as a result of reduction in repulsive forces between ether groups in PEG and surface of the membrane.

Overall, these results suggest that there is an association for tested vanillin concentrations up to 2.3 g/L, between improvement of permeability and the enhancement of hydrophilicity, caused by physical surface modification of the UH004 P membrane with vanillin. For higher vanillin concentrations, improvement of permeability is observed despite the lower hydrophilicity; further research is needed to explain this inconsistency. The formation of a complex between vanillin and membrane could, for instance, be one reason for improvement of the PEG solution flux [35].

Table 5 illustrates the effect of modification on the PEG rejection for various vanillin concentrations.

In general, a clear benefit of vanillin adsorption in the enhancement of PEG rejection could not be identified in this analysis. However, only trace amounts of improvement in vanillin concentrations below 1.3 g/L were observed. This improvement might be related to the pore narrowing due to the adsorption of feed solutes both on the membrane surface and pore walls as well. It should also be noted that the inherent trade-off between permeability and rejection emerges specifically at the highest exposure concentration (2.8 g/L) and caused about 37 % improvement in permeability of PEG solution, and about 5 % reduction in PEG rejection.

### 3.4. The Quantity of Vanillin Remaining in the Membrane Structure after Experiments

**Figure 6:**
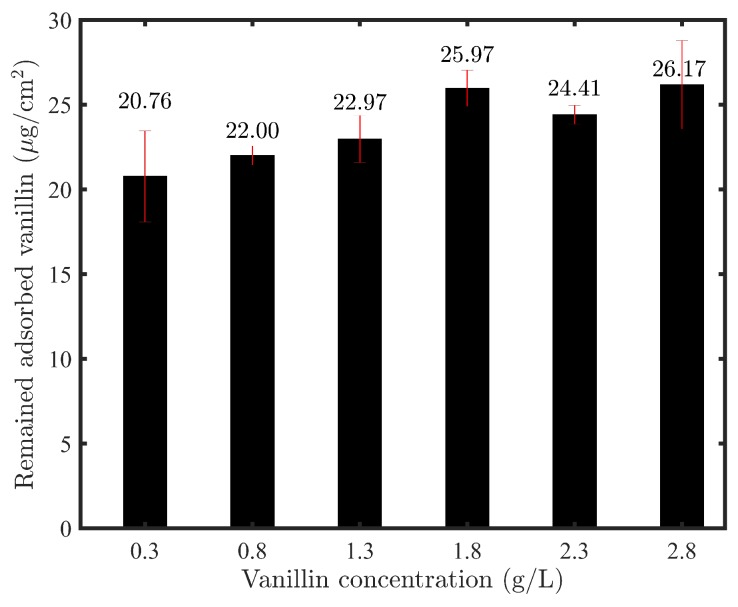
The amount of vanillin remaining on the surface and structure of modified membranes at the end of experiments.

At the end of the experiments, the amount of vanillin remaining on the surface and structure of membranes was measured by extraction and UV analysis, and the averaged results are shown in Figure 6.

As can be seen from Figure 6, 20–26 μg/cm${}^{2}$ vanillin at pH 3.8 remains on the surface and structure of the membrane, which is in line with Virtanen et al.’s findings at this pH [36]. Hence, it could conceivably be hypothesized that this range is the equilibrium state of adsorbed vanillin regardless of the concentration which is used for the modification.

Although the physical modification suffers from loss of adsorbed layer during long-term operation, the presence of vanillin on the feed solution can reduce the bacterial growth, and biofilm formation on the surface of the membrane due to its quorum quenching mechanisms. The vanillin concentration of 63 to 250 μg/mL was demonstrated to possess an inhibition activity against biofilm formation of Aeromonas hydrophilia [13]. In our previous study, approximately 250 μg/cm${}^{2}$ vanillin adsorbed on the surface of the membrane when the membrane was modified with 1.25 g/L vanillin [18]. Therefore, at the highest concentration (2.8 g/L) in this study, it can be hypothesized that the release of vanillin to the feed and especially to the area close to the surface of the membrane has the potential to reduce the bacterial growth and thus biofilm formation on the surface of the membrane.

### 3.5. The Influence of Modification on the Membrane Performance in the Filtration of Wood Extract

**Figure 7:**
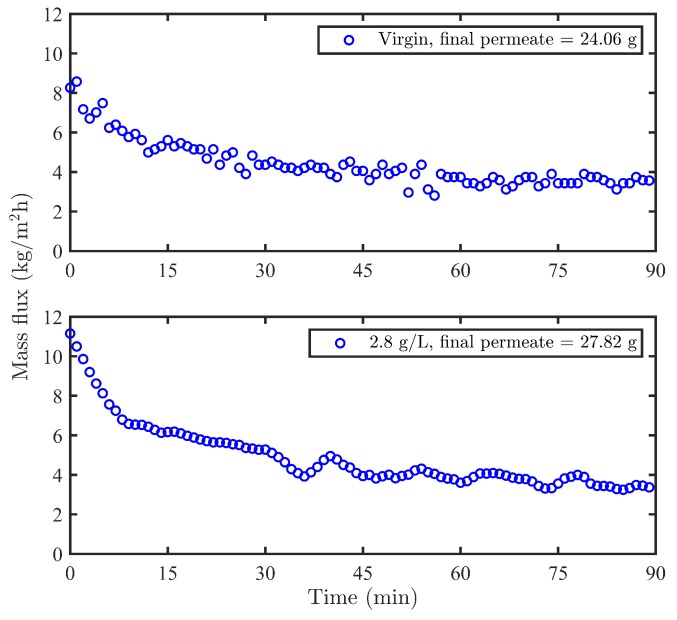
The effect of modification on the wood extract permeance.

The maximum PEG solution permeability (Table 3) and vanillin adsorption on the surface of the membrane (Figure 4) were achieved when the commercial membrane was modified at 2.8 g/L vanillin concentration. Thus, this concentration was selected in order to evaluate the effect of modification on the wood extract permeance and rejection. The modification in this concentration was repeated thrice to check the reliability of measurements. A minor observed improvement of wood extract flux in modified membranes with 2.8 g/L vanillin (Figure 7) can be ascribed to the role of vanillin in slightly increasing the hydrophilicity of the modified membranes compared to the virgin one. Table 6 shows the TOC and lignin rejection analysis for virgin membrane and the modified membrane with 2.8 g/L vanillin, as well as their final collected permeate mass and relative steady permeate flux (RSPF). It can be seen that the lignin rejection for modified membrane (2.8 g/L vanillin) declined by 12–28% compared to virgin membrane. The possible interference of vanillin in lignin UV absorption measurement was evaluated to be minor as the UV absorbance would increase only about 3% if all the adsorbed vanillin would be released to the permeate, and thus it didn’t affect the UV results of lignin at 280 nm significantly.

The pure water flux before static adsorption of vanillin and after membrane fouling (wood extract filtration) was measured (Table 7), and it was noticed that the modified membrane (2.8 g/L) possessed lower flux reduction when compared to the virgin membrane, indicating better antifouling features of the modified membrane. In addition, the similarity between pure water flux before adsorption of vanillin for modified membrane and virgin membrane eliminates the effect of initial membrane properties on experimental results. The lower fouling proneness of modified membrane can enhance the separation of the lignin from the other organic compounds (i.e., lower lignin rejection, Table 6).

## 4. Conclusions

Surface of commercial PES membrane was modified by controlled adsorption of vanillin to minimize the membrane fouling phenomenon and to improve the membrane performance by increasing the hydrophilicity of the membrane. The presence of vanillin and its increasing adsorption potential trends in higher vanillin concentrations were clearly confirmed by observable changes in FTIR spectra after modification. Membranes with better PEG solution permeabilities and hydrophilicities were obtained as a result of this modification while the rejection remained almost constant. In the best case scenario in terms of membrane performance, the modified membrane showed better antifouling characteristics and lignin separation compared to the virgin membrane. In addition, according to literature findings, the release of vanillin from the surface of the membrane may also interrupt the bacterial growth and hence reduce the biofilm formation on the surface of the membrane. Further research on the incorporation of natural or synthesis quorum sensing inhibitory agents, such as vanillin within the membrane structure would be a useful way of developing the next generation of antifouling membranes. This incorporation could offer valuable insights into prevention of biofilm formation on the surface of the membrane, which is considered as one of the major impediments in the application of membrane bioreactor and reverse osmosis membranes.

## Figures and Tables

**Table 1:** The FTIR peaks of glycerol as UH004 P preservative agent.

This Study (cm${}^{-1}$)	Literature (cm${}^{-1}$)	Peak Assignments
923	920 [26], 923 [22]	O–H bending
1039	1037 [22]	alcoholic C–O asymmetric stretching vibration
1443–1104	1100–450 [26]	C–O stretching from secondary and primary alcohols
1655	1650 [26], 1647 [22]	H${}_{2}$O bending
1462–1400	1400–460 [26]	C–O–H bending
2879 and 2931	2880 and 2930 [26]	C–H stretching
3313	3313 [22]	O–H stretching

**Table 2:** Main functional groups in commercial membrane UH004 P [27,28,29].

FTIR-Peaks (cm${}^{-1}$)	Peak Assignments
1103	S=O stretching vibration
1146	Symmetric SO${}_{2}$ stretches of sulfone group
1235	Aromatic ether band
1289	C–N stretch (PVP)
1321	Asymmetric SO${}_{2}$ stretches of sulfone group
1423	CH${}_{2}$ bending (PVP)
1486 and 1578	Aromatic bands (characteristics for PES)
1671	C=O carbonyl group (PVP)
2850–2856	γs CH${}_{2}$ symmetric aliphatic stretch (PVP)
2876	γs CH${}_{3}$ symmetric aliphatic stretch (PVP)
2925	γa CH${}_{2}$ asymmetric aliphatic stretch (PVP)
2952	γa CH${}_{3}$ asymmetric aliphatic stretch (PVP)
3096 and 3069 (broad band)	CH–aromatic stretch
3200–3600	Hydrogen bonded OH band (PVP)

**Table 3:** Improvement (%) of PEG solution permeabilities after exposure of membranes to different vanillin concentrations at the pH 3.8.

Vanillin Concentration (g/L)	Test 1 (%)	Test 2 (%)	Test 3 (%)	Average (%)	Standard Deviation
0.0	−3.70 −5.30	−4.54 −6.72	−11.19 –	−6.29 *	2.96
0.3	0.60	0.57	0.55	0.57 *	0.03
0.8	2.34	3.71	2.67	2.91 *	0.72
1.3	27.39	21.08	21.40	23.29 *	3.55
1.8	22.11	14.54	–	18.33 *	5.35
2.3	20.73	16.27	–	18.50 *	3.15
2.8	38.83	35.90	–	37.37 *	2.07

* Statistically significant correlation between vanillin concentration and improvement (%) of PEG solution permeability using one-tailed *t*-tests (*p*-value < 0.05) with a 95% confidence level.

**Table 4:** The effect of precleaning with Ultrasil 110 cleaning agent and vanillin adsorption on the contact angle of UH004 P membranes (error is based on 95% CI).

Samples	Contact Angle (°)
UH004 P and preservative agent	32.08 ± 0.61
UH004 P (precleaned with Ultrasil 110 cleaning agent)	42.74 ± 1.68
UH004 P reference (water at pH 3.8) *	41.94 ± 1.86
0.3 g/L vanillin *	40.43 ± 0.49
0.8 g/L vanillin *	33.40 ± 0.58
1.3 g/L vanillin *	29.49 ± 2.05
1.8 g/L vanillin *	29.17 ± 1.90
2.3 g/L vanillin *	29.88 ± 0.66
2.8 g/L vanillin *	39.67 ± 0.74
4.8 g/L vanillin	39.14 ± 1.08

* Statistically significant correlation between contact angle and vanillin concentration using two-tailed *t*-tests (*p*-value < 7.07 × 10${}^{-6}$) with a 95% confidence level.

**Table 5:** PEG rejection before and after exposure of membranes to different vanillin concentrations at the pH 3.8.

Vanillin Concentration (g/L)	PEG Rejection (%) before Adsorption				PEG Rejection (%) after Adsorption			
	Test 1	Test 2	Test 3	Average	Test 1	Test 2	Test 3	Average
0	87	85	90	87	86	86	90	87
0.3	92	91	91	91	94	92	92	93
0.8	93	91	88	91	95	93	90	93
1.3	89	87	91	89	90	89	90	90
1.8	85	83	–	84	75	85	–	80
2.3	82	90	–	86	78	90	–	84
2.8	88	72	–	80	81	68	–	75
0	92	86	–	89	86	89	–	88

**Table 6:** TOC (total organic carbon) rejection, lignin rejection, final collected permeate mass, and RSPF (relative steady permeate flux) of virgin membrane and modified one with 2.8 g/L vanillin (the average of three replicate tests).

Samples	R${}_{\mathrm{TOC}}$ (%)	R${}_{\mathrm{lignin}}$ (%)	Final Permeate (g)	RSPF at Last 10 min (kg/m${}^{2}$h)
Virgin	76.12	42.32	24.06	3.00
2.8 g/L	74.56 ± 1.02	34.11 ± 3.38	24.92 ± 3.3	3.18 ± 0.27

**Table 7:** Pure water flux before static adsorption of vanillin and after wood extract filtration for unmodified/modified UH004 P membranes.

Samples	Pure Water Flux (kg/m${}^{2}$h)		PWF${}_{R}$ (%)
	before Filtration	after Filtration	
Virgin	62.44 ± 0.12	28.92 ± 3.20	53.68 ± 5.20
2.8 g/L	61.90 ± 0.57	35.91 ± 1.33	41.99 ± 1.88

## Supplementary Materials

The following are available online at https://www.mdpi.com/2077-0375/9/4/56/s1, Figure S1: PEG solution permeabilities before and after modification with vanillin. Polyethersulfone membranes (UH004 P) were modified by different concentration of vanillin (0.3 g/L, 0.8 g/L and 1.3 g/L) and each experiment was repeated thrice. Figure S2: PEG solution permeabilities before and after modification with vanillin. Polyethersulfone membranes (UH004 P) were modified by different concentration of vanillin (1.8 g/L, 2.3 g/L and 2.8 g/L) and each experiment was repeated twice. Figure S3: The UV absorption of vanillin in different concentrations at wavelength of 308 nm and pH 5.6.
