# Supplementary material for: Vanillin as an Antifouling and Hydrophilicity Promoter Agent in Surface Modification of Polyethersulfone Membrane
Source: Membranes (Basel). 2019 Apr 24;9(4):56. doi: 10.3390/membranes9040056 (PMC6523077; doi:10.3390/membranes9040056)
Supplement: Supplementary file 1 [file membranes-09-00056-s001.pdf]

membranes

MDPI

Article

Supplementary Material: Vanillin as an Antifouling and Hydrophilicity Promoter Agent in Surface Modification of Polyethersulfone Membrane

Mohammadamin Esmaeili[1]*, Tiina Virtanen [1], Jussi Lahti [1,2], Mika Mänttäri [1] and Mari Kallioinen [1,2]

1 LUT School of Engineering Science, Department of Separation and Purification Technology, Lappeenranta University of Technology; tiina.virtanen@lut.fi (T.V.); jussi.lahti@lut.fi (J.L.); mika.manttari@lut.fi (M.M.)
2 LUT Re-Source Platform, Lappeenranta University of Technology, P.O.Box 20, 53851 Lappeenranta, Finland; mari.kallioinen@lut.fi (M.K.)
* Correspondence: mohammadamin.esmaeili@lut.fi; Tel.: +358-44-968-3768

Received: 8 March 2019; Accepted: 18 April 2019; Published: 24 April 2019

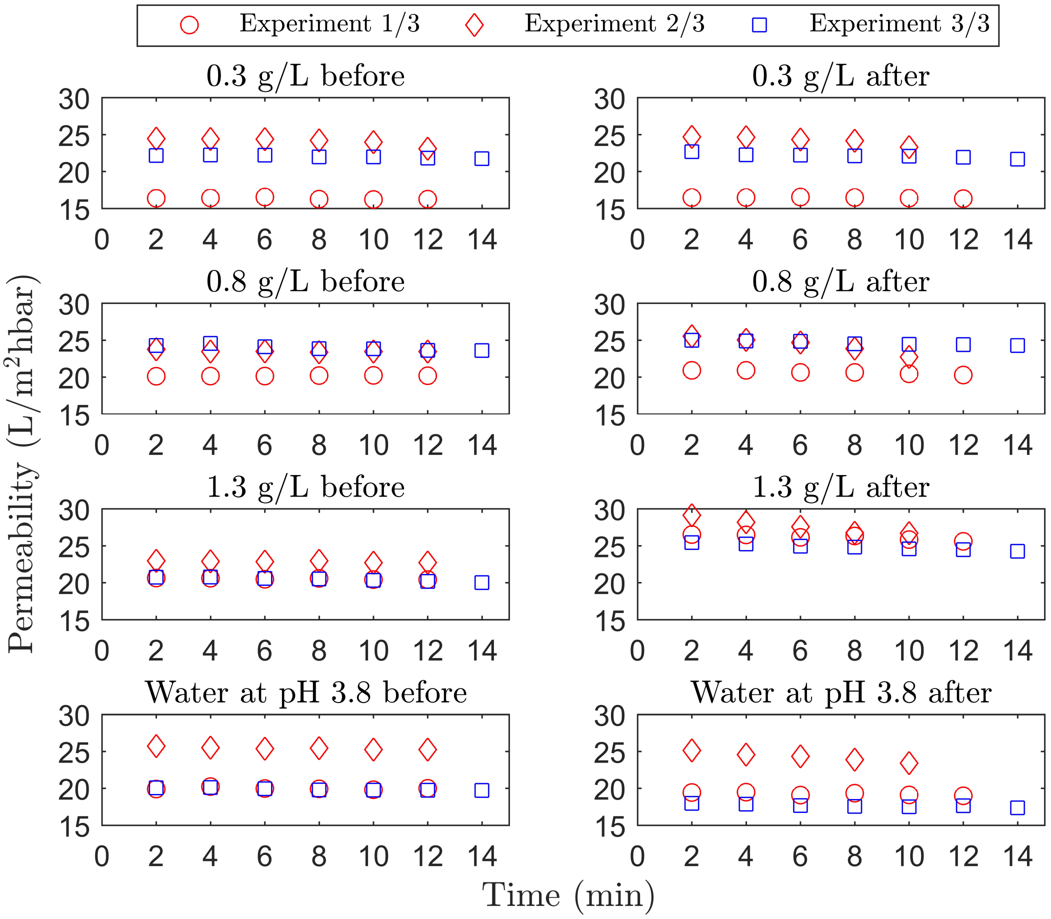

Figure S1. PEG solution permeabilities before and after modification with vanillin. Polyethersulfone membranes (UH004 P) were modified by different concentration of vanillin (0.3 g/L, 0.8 g/L and 1.3 g/L) and each experiment was repeated thrice.

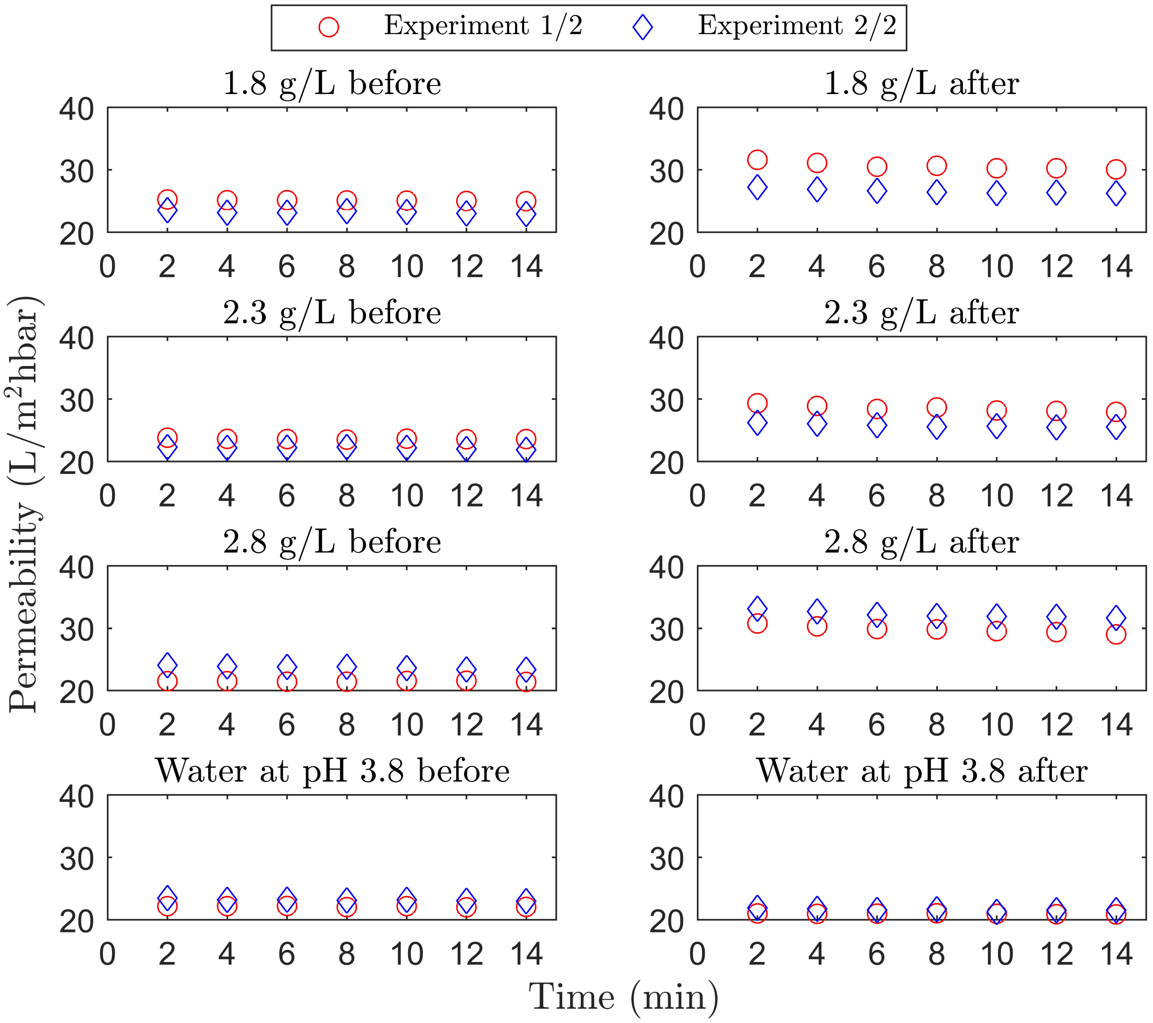

Figure S2. PEG solution permeabilities before and after modification with vanillin. Polyethersulfone membranes (UH004 P) were modified by different concentration of vanillin (1.8 g/L, 2.3 g/L and 2.8 g/L) and each experiment was repeated twice.

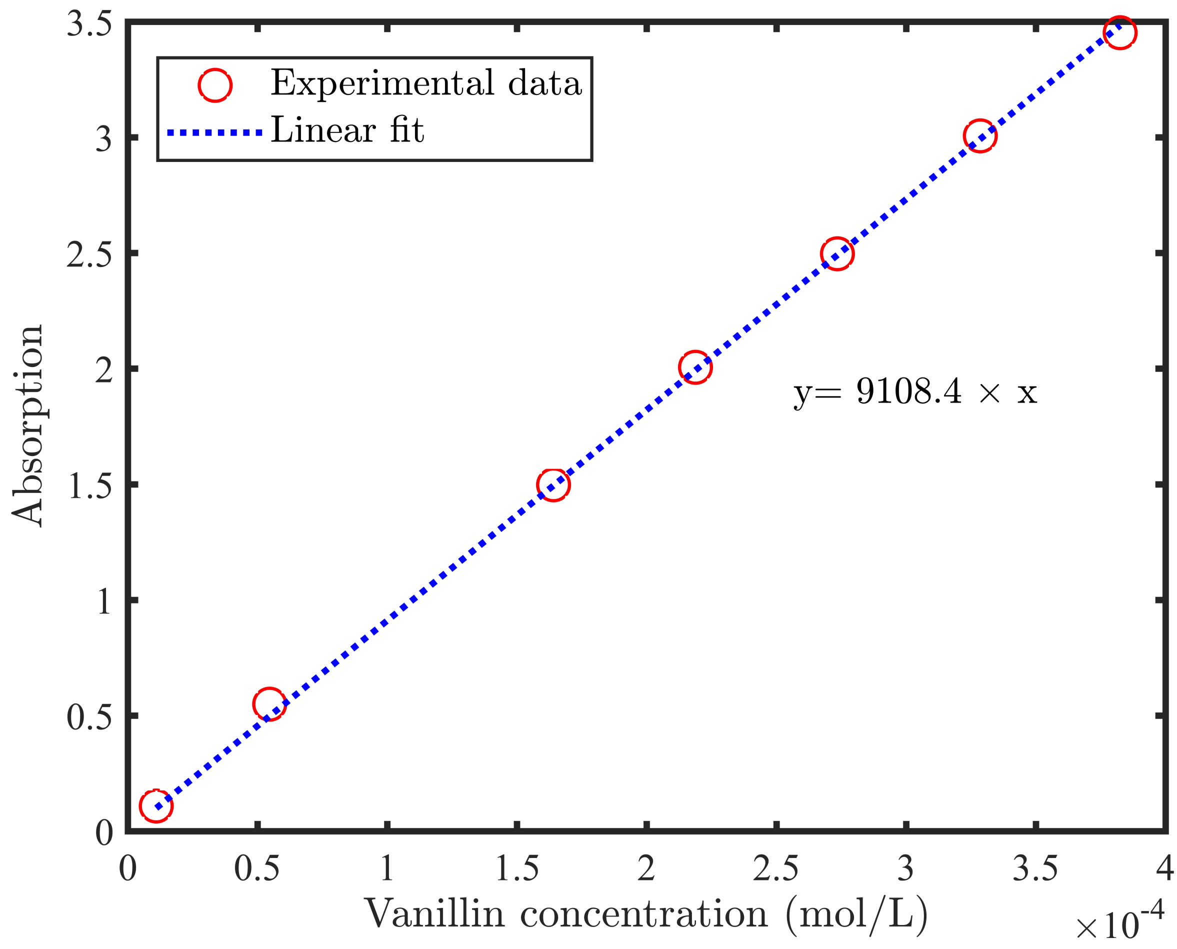

Figure S3. The UV absorption of vanillin in different concentrations at wavelength of 308 nm and pH 5.6.

© 2019 by the authors. Licensee MDPI, Basel, Switzerland. This article is an open access article distributed under the terms and conditions of the Creative Commons Attribution (CC BY) license (http://creativecommons.org/licenses/by/4.0/).
